# Dynamic Evolution and Correlation between Metabolites and Microorganisms during Manufacturing Process and Storage of Fu Brick Tea

**DOI:** 10.3390/metabo11100703

**Published:** 2021-10-13

**Authors:** Jing Li, Ran Xu, Lixuan Zong, Joseph Brake, Lizeng Cheng, Jie Wu, Xiaobin Wu

**Affiliations:** 1 Development Center of Plant Germplasm Resources, College of Life Sciences, Shanghai Normal University, Shanghai 200234, China; lijing52@shnu.edu.cn (J.L.); xu_ran0801@wuxiapptec.com (R.X.); zonglixuan0829@163.com (L.Z.); wj18133673372@163.com (J.W.); 2 Department of Biochemistry and Redox Biology Center, University of Nebraska-Lincoln, Lincoln, NE 68588, USA; brakejoseph@gmail.com; 3 School of Agriculture and Biology, Shanghai Jiao Tong University, 800 Dongchuan Road, Shanghai 200240, China; dr_cheng@sjtu.edu.cn

**Keywords:** Fu brick tea, bioprocesses, microbiology, metabolomics, bioactivity

## Abstract

Fu brick tea (FBT) is one of the major brands of dark tea. Microbial fermentation is considered the key step in the development of the special characteristics of FBT. The systemic corelationship of the microbiome and metabolomics during manufacture of Fu brick tea is not fully understood. In this study, we comprehensively explored the microbiome and metabolite dynamic evolution during the FBT manufacturing processes, and revealed decisive factors for the quality and safety of FBT based on the grouped methods of metabolomics combined with biochemical measurements, microbiome sequencing combined with quantitative polymerase chain reaction (PCR), and multiplex analysis. Both the microbiome and quantitative PCR showed that fungi displayed concentrated distribution characteristics in the primary dark tea samples, while bacterial richness increased during the flowering processes and ripening period. All microorganism species, as well as dominant fungi and bacteria, were identified in the distinct processes periods. A total of 178 metabolites were identified, and 34 of them were characterized as critical metabolites responsible for metabolic changes caused by the corresponding processes. Metabolic analysis showed that most metabolites were decreased during the FBT manufacturing processes, with the exception of gallic acid. Multivariate analysis verified that the critical metabolites were correlated with specific dominant microbial species. All the top fungal species except *unclassified_g_ Aspergillus* showed positive correlations with six critical metabolites (L-The, epigallocatechin (EGC), Gln, tea polyphenol (TP), tea polysaccharides (TPs) and caffeine). Five of the top bacteria species (*Cronobacter*, *Klebsiella*, *Pantoea*, *Pluralibacter,* and *unclassified_ f_Entero-bacteriaceae*) showed positive correlations with epigallocatechins and tea polyphenols, while the other 11 top bacterial species correlated negatively with all the critical metabolites. The content of amino acids, tea polyphenols, tea polysaccharides, and flavonoids was reduced during microbial fermentation. In conclusion, our results reveal that microbial composition is the critical factor in changing the metabolic profile of FBT. This discovery provides a theoretical basis for improving the quality of FBT and enhancing its safety.

## 1. Introduction

Fu brick tea (FBT) is a typical dark tea manufactured by microbial fermentation of *C. sinensis* tea leaves, which are also the raw material of green tea [1]. FBT was invented during the Ming dynasty, and is mainly produced in the Hunan province of China [2]. Its popularity is due to its attractive flavor and putative health benefits, including antiobesity, antioxidative, antihyperlipidemic, and antimicrobial properties [1].

The main manufacturing processes of FBT are steaming, piling, pressing, fermentation, and drying [3]. There is also a special process called “flowering”, which is actually the incubation of the spores of a special fungus. The interior of FBT is widely interspersed with small golden-brown spots, which are the ascocarp of *Eurotium cristatum*, and are judged as a quality standard of FBT. This process is associated with a series of reactions, such as degradation, oxidation, condensation, structural modification, methylation, and glycosylation. Previous studies have reported that the content of catechin derivatives, flavonoids and their glycosides, phenolic acids, alkaloids, and terpenoids are significantly changed during fermentation processes [4]. The complex biochemical profiles of FBT are considered critical elements in the development of its special characteristics, and are related to the microbiome during FBT manufacturing processes. Both fungi and bacteria have been identified using culture-dependent and culture-independent methods as important members of the microbial community for the production of FBT [5,6].

For example, *Eurotium cristatum* has been identified as the predominant fungal species during the processing of FBT by culture-dependent and multilocus sequence typing methods [7,8]. Meanwhile, Fu et al. found that *Lactococcus* was the predominant bacterial genus in FBT [9]. However, in these studies, only the fungi or bacteria communities and a small number of common compounds were characterized. Systematic studies between the microbiome and metabolites are limited. Therefore, a more comprehensive investigation is needed to understand and clarify the dynamic evolution of the microbiome and metabolites during the entire manufacturing process. High-throughput sequencing is a powerful tool to explore the richness and species of microorganisms [10]. At the same time, quantitative PCR (qPCR) is regarded as an accurate method for the detection and quantification of microorganisms due to its specificity, high sensitivity, rapidity, and diversity [11]. To precisely investigate the comprehensive and dynamic variety of microorganisms during the processing and storage of Fu brick tea, we combined high-throughput sequencing and qPCR to directly and precisely quantify copy numbers of microbial genome for different samples.

The metabolomics strategy can unbiasedly characterize or quantify as many of the compounds present in a biological sample as possible [12], and allow us gain an insight into the biochemical changes that occur with time and processing [13]. A metabolomics approach has been applied to the investigation of biochemical composition changes during the microbial fermentation processes [14], and characterized the dynamic changes in metabolite profile and taste characteristics of FBT [15]. The multivariate correlation analysis of metabolomics data in FBT research shows that the metabolomics approach is a powerful tool to elucidate biochemical changes occurring during the manufacturing processes. The HPLC–MS approach has been widely utilized in metabolomics, owing to its high-throughput capacity, powerful separation ability, high resolution, and strong sensitivity [16]. This approach mainly focuses on the identification of small molecular endogenous metabolites in organisms [17]. However, the nutritional and healthy ingredients in Fu brick tea are mostly macromolecules, such as polysaccharides, tea polyphenols, and catechin [18]. To make up for this defect, biochemical methods were used to assess these contents of macromolecules in Fu brick tea.

In this paper, teas from various stages of the manufacturing processes were selected: primary dark tea, middle stage of piling-fermentation of tea material, later stage of piling-fermentation of tea material, flowering 6 days, flowering 9 days, ripened 180 days, and ripened 540 days. All tea samples were selected to study the shift in the microbial community structure, and the correlation between the microbiome and metabolites during FBT processing. A potent metabolomics tool was used to characterize hundreds of endogenous metabolite changes during the fermentation processes. We applied microbiome sequencing combined with multivariate analysis to elucidate the correlation of metabolic profiles and microbial community, and explored the changes in functional compounds during the manufacturing processes. This study provides deep insight into evaluating the complex relationship between the functional microorganisms, metabolites, and dominant functional compounds in FBT. 

## 2. Results 

### 2.1. Sequence Statistics of Fungi and Bacteria of Fu Brick Tea Samples 

To comprehensively investigate the dynamic evolution of microbes during manufacturing process and storage of Fu brick tea, specific regions of fungal ITS gene and bacterial 16s rDNA were cloned by PCR with specific primers. These PCR fragments were sequenced by high-throughput sequencing (Illumina sequence). The results exhibited that the total number of qualified fungal raw sequences for Fu brick tea samples was 13,780,204 (Table 1). By comparison, 1,055,795 qualified bacterial sequences were obtained from Fu brick tea samples (Table 2). The total sequence lengths of fungi and bacteria were 312,864,617 bp and 439,213,816 bp, respectively. The average bacterial sequence length of 416.2 bp was higher than the fungal sequence length of 226.9 bp. 

### 2.2. Comparisons of Fungal Community and Richness during the Manufacturing Process and Storage 

**Figure 1:**
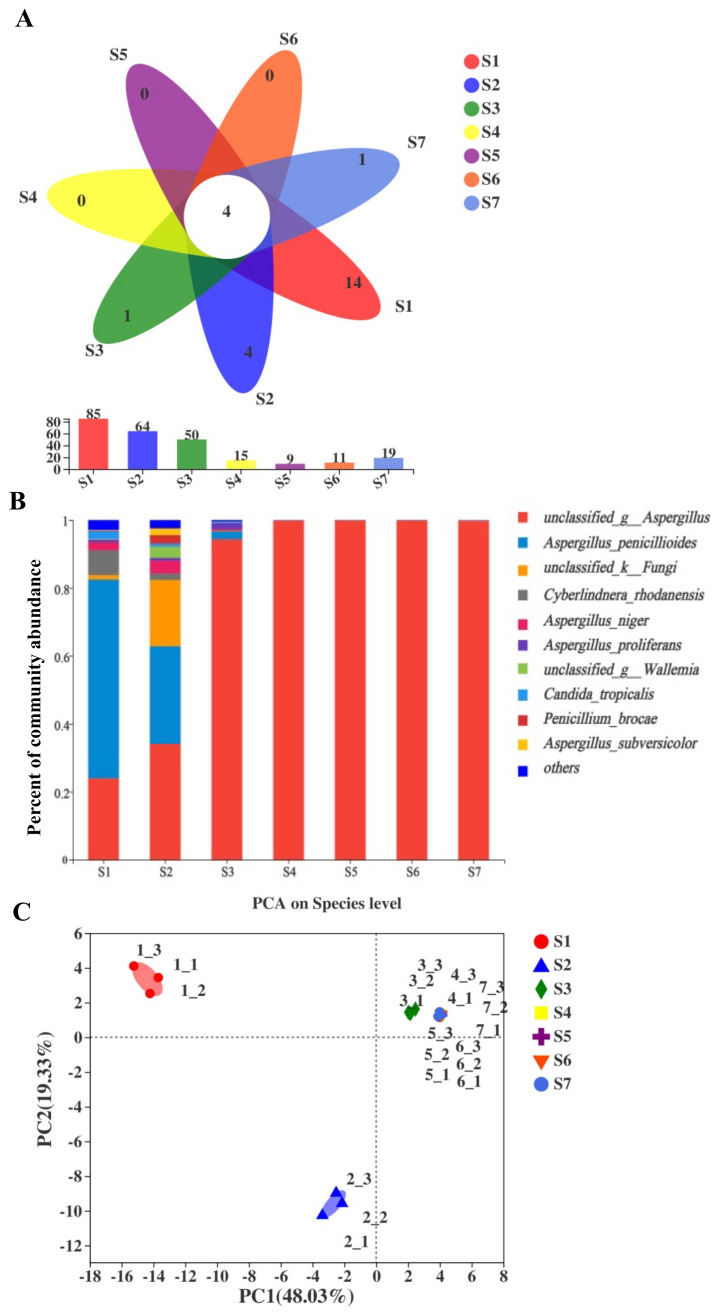
Comparisons of fungal composition and richness in different samples of Fu brick tea. (**A**) Venn diagram analysis of fungal OTU level in samples during manufacturing process of Fu brick tea. (**B**) Fungal taxonomic compositions showing the fugal successions at species level. The fungal communities in eight period samples are comparable diverse. (**C**) Principal component analysis (PCA) of fungal communities in 7 FBT samples at the species level. (note: The taxonomic abundance <1% were classified into “others”).

Most of the microorganisms in Fu brick tea comprised fungi and bacteria. The quality-filtered fungal sequences of all seven FBT samples were clustered into 138 fungal OTUs at the 97-similarity cut-off. The rarefaction curve (Figure S1A) and Shannon curves (Figure S1B) had already reached a plateau at this sequencing depth, suggesting that the sequencing was adequately deep. The Shannon index, Simpson index, Chao1 index, and accumulated cyclone energy (ACE) index values are exhibited in Table S1. Shannon and Simpson diversity index results indicated that fungal diversity significantly increased between sampling points S1 and S2, but remained relatively constant from S4 until the end of the manufacturing processes (Figure S2). The Chao1 and ACE index values indicated that species richness gradually decreased from S2 to S5 of the FBT manufacturing processes, and the lowest OTU number appeared at sampling point S5 (Table S1). We used Venn diagram analysis to show the shared OTUs of fungi during different stages of the manufacturing processes (Figure 1A). The number of fungal OTUs was decreased during the fermentation processes, and increased during the ripened stage. The seven FBT samples shared four fungal OTUs. The highest OTU number (85) appeared in the S1 sample, but the OTU number gradually decreased to 9 in the S5 sample. When the tea was ripened, the fungal OTU number was increased to 19 in the S7 sample.

To compare the fungal communities, the fungal taxonomic compositions were identified across all stages of the manufacturing processes. The 138 fungal OTUs could be assigned into 2 different phyla, 5 classes, 8 orders, 8 families, 8 genera, and 13 species. *Aspergillus* was the predominant genus in all samples, accounting for 90% of total effective sequences (Figure 1B). *Aspergillus-penicillioide* and some other fungi species were identified in S1 and S2 samples, but disappeared in the later stages of the manufacturing processes. 

At the species level, *unclassified_g_Aspergillus* was the most dominant fungal species, constituting 23.99–99% of all sequences in each sample (Figure 1B). While *Aspergillus-penicillioide* was the predominant species in the raw material sample (S1), its relative abundance dramatically decreased from 58.48% in S1 to 2.13% in S3. Moreover *Aspergillus-penicillioide* was undetected across the entire the later fermentation processes (S4–S7). *Aspergillus_niger* was dominant in S1 (2.39%), then increased to 3.47% in S2, but decreased to less than 1% in the S3 sample. *Penicillium brocae* and *unclassified_g_Wallemi* accounted for 0.17% and 0.23% of all sequences, respectively, in sample S1, and then increased to 2.53% and 3.34% of all sequences, respectively, in S2. However, in both cases, the relative abundance then dramatically decreased to undetected in S3. *Aspergillus subversicolor* and *unclassified-k-Fungi* only accounted for 0.18% and 1.31% of all sequences, respectively. Subsequently, the relative abundance gradually decreased to undetected after S3 (Figure 1B). 

To assess β-diversity, PCA analysis was used to investigate overall differences in fungal community structure among the samples across the FBT manufacturing processes. The results indicated that the samples could be separated into three periods based on Euclidean distance and Bray–Curtis dissimilarity analyses, designated stage I (S1), stage II (S2), and stage III (S3–S7) (Figure 1C).

### 2.3. Comparisons of Bacterial Community and Richness during the Manufacturing Processes and Storage

Another main microorganism found in Fu brick tea was bacteria. The quality-filtered bacterial sequences of all seven samples were clustered into 1091 OTUs at the 97-similarity cut-off. The rarefaction curve (Figure S3A) and Shannon curves (Figure S3B) had already reached a plateau at this sequencing depth, suggesting that the sequencing was adequately deep. Shannon, Simpson, Chao1, and ACE index values are exhibited in Table S2. Shannon and Simpson index analyses indicated that bacterial diversity was fluctuant, but the highest diversity of bacteria appeared in the ripened 18 months (S7) sample (Figure S4). The Chao1 and ACE index values indicated that species richness gradually increased from the S1 to S4 course of the FBT manufacturing processes, reaching the lowest OTU number at sampling point S6, followed by an increase until the end of the manufacturing processes. 

**Figure 2:**
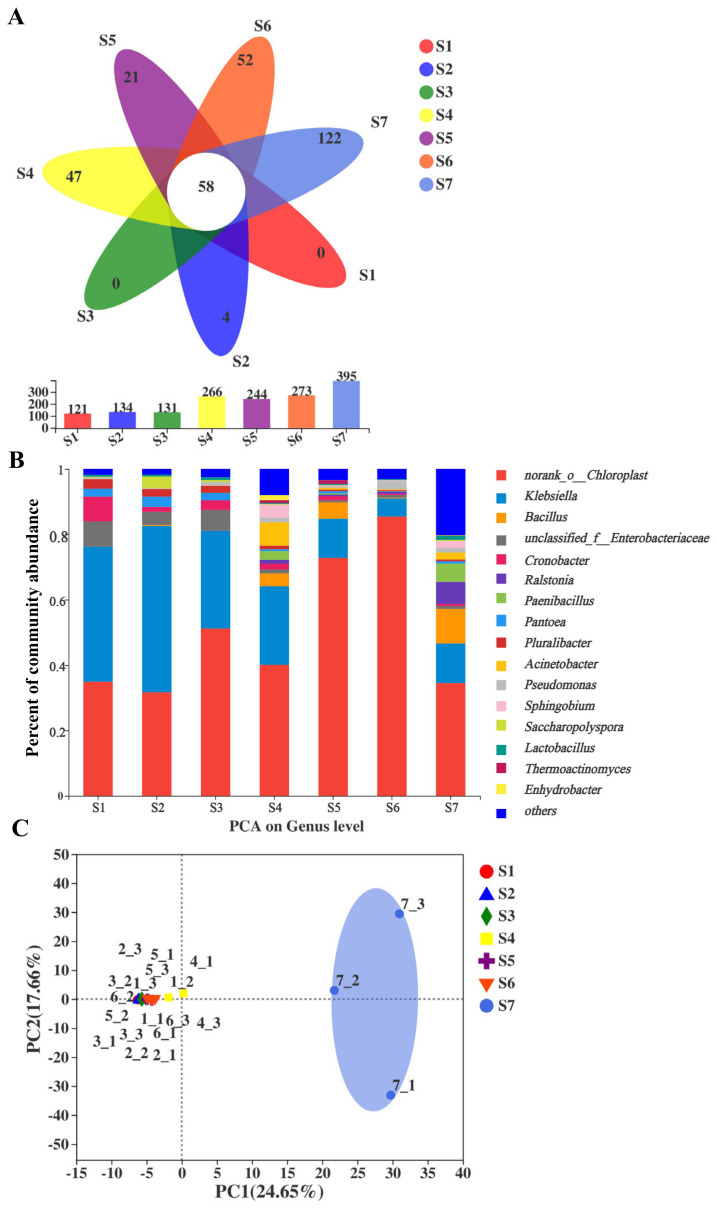
Comparisons of bacterial composition and richness in different samples of Fu brick tea. (**A**) Venn diagram analysis of bacterial OTU level in samples during manufacturing process Fu brick tea. (**B**) Bacterial taxonomic compositions showing the bacterial successions at species level. The bacterial communities in eight period samples are comparable diverse. (**C**) Principal component analysis (PCA) of bacterial communities in 7 FBT samples at the species level. (note: The taxonomic abundance <1% were classified into “others”).

In our study, a total of 1091 bacterial OTUs were identified and assigned into 31 different phyla, 65 classes, 162 orders, 296 families, 577 genera, and 1091 species. Venn diagram analysis was used to clarify the bacterial diversity during different stages of the manufacturing processes (Figure 2A). The number of bacterial OTUs was increased during the fermentation processes and ripened stages. The seven FBT samples shared 58 bacterial OTUs. The total OTU number gradually increased from 121 to 395 during the manufacturing processes, with the highest OTU number in the S7 sample. *Klebsiella* was the predominant genus in S1 samples, accounting for 41.25% of total effective sequences, but it sharply decreased to 5.52% in S6. The *norank_o_Chloroplast (Cyanobacteria)* was the most dominant across the samples, with the highest richness (85.44%) appearing in S6, which then decreased to 34.5% in S7. *Pantoea* and *Pluralibacter* remained 2–3% in the S1, and S3 samples, but disappeared in S4. Additionally, *Pantoea* and *Pluralibacter* were found in the later stages of the manufacturing processes. *Saccharopolyspora* richness was increased from 0.27% in S1 to 3.53% in S2, then dramatically decreased to undetected in S3. *Paenibacillus* and some other bacteria increased in S4, but decreased in the S5 and S6 periods, then reemerged in S7 (Figure 2B).

To assess β-diversity, PCA analysis was used to investigate overall differences in bacterial community structure among the samples across the FBT manufacturing processes. The results indicated that the samples could be classified into two periods based on Euclidean distance and Bray–Curtis dissimilarity analyses, designated stage I (S1–S6) and stage II (S7) (Figure 2C).

### 2.4. Quantitative PCR (qPCR) Detection of Fungi and Bacteria during Processing and Storage of Fu Brick Tea

**Figure 3:**
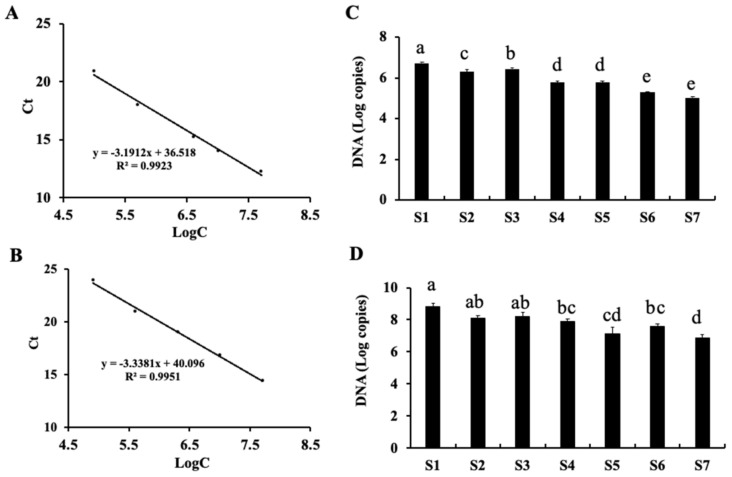
Quantitative changes of fungi and bacteria detected by qPCR during the processing and storage of Fu brick tea. (**A**) Standard curve of fungal plasmid; (**B**) Standard curve of bacterial plasmid; Quantitative changes of fungi (**C**) and bacteria (**D**) during processing and storage of Fu brick tea.

Real-time quantitative PCR (qPCR) is regarded as an accurate method for the detection and quantification of microorganisms, owing to its high sensitivity, specificity, rapidity, and diversity. To make up for the relative copy number feature of high-throughput sequencing, we utilized qPCR to directly quantify copy numbers of the microbial genome for different samples. Firstly, we constructed standard plasmids for generation of fungal or bacterial standard curves. The 188 bp fragment (091-279) of yeast (BY4741, Saccharomyces cerevisiae) ITS gene cloned by PCR with specific primers was inserted into Bam HI and Xho I sites in the p416-TEF vector for expression in yeast. The 659 bp fragment (031-690) of *E. coli* 16s rDNA obtained by PCR with specific primers was inserted into Bam HI and Xho I sites in the p416-TEF vector. The p416-TEF-ITS(188) and p416-TEF-16S rDNA(659) were utilized for generation of fungal and bacterial standard curves, respectively. The fungal standard curve (Figure 3A) and bacterial standard curve (Figure 3B) were generated by a cycle threshold (Ct) value obtained from 10-fold serial dilutions of plasmid DNA in deionized water to produce five different concentrations. Genomic DNAs were extracted from different period samples of Fu brick tea as templates, and quantitative PCR was conducted with the same primer sets as those used for the standard curve qPCR. The abundance of bacteria and fungi showed a decreased trend during the processing and storage of Fu brick tea (Figure 3C,D). The copy numbers of fungi were 10^5^–10^7^ copies/μL (Figure 3C), and bacterial copy numbers were 10^5^–10^9^ copies/μL (Figure 3D). The abundance of bacteria was significantly higher than that of fungi. These results were consistent with the overall trends of fungi and bacteria revealed by high-throughput sequencing. Our results indicated that the abundance of bacteria and fungi were decreased during the processing and storage of Fu brick tea.

### 2.5. Metabolomics Analysis of Fu Brick Tea Samples during Processing and Storage

**Figure 4:**
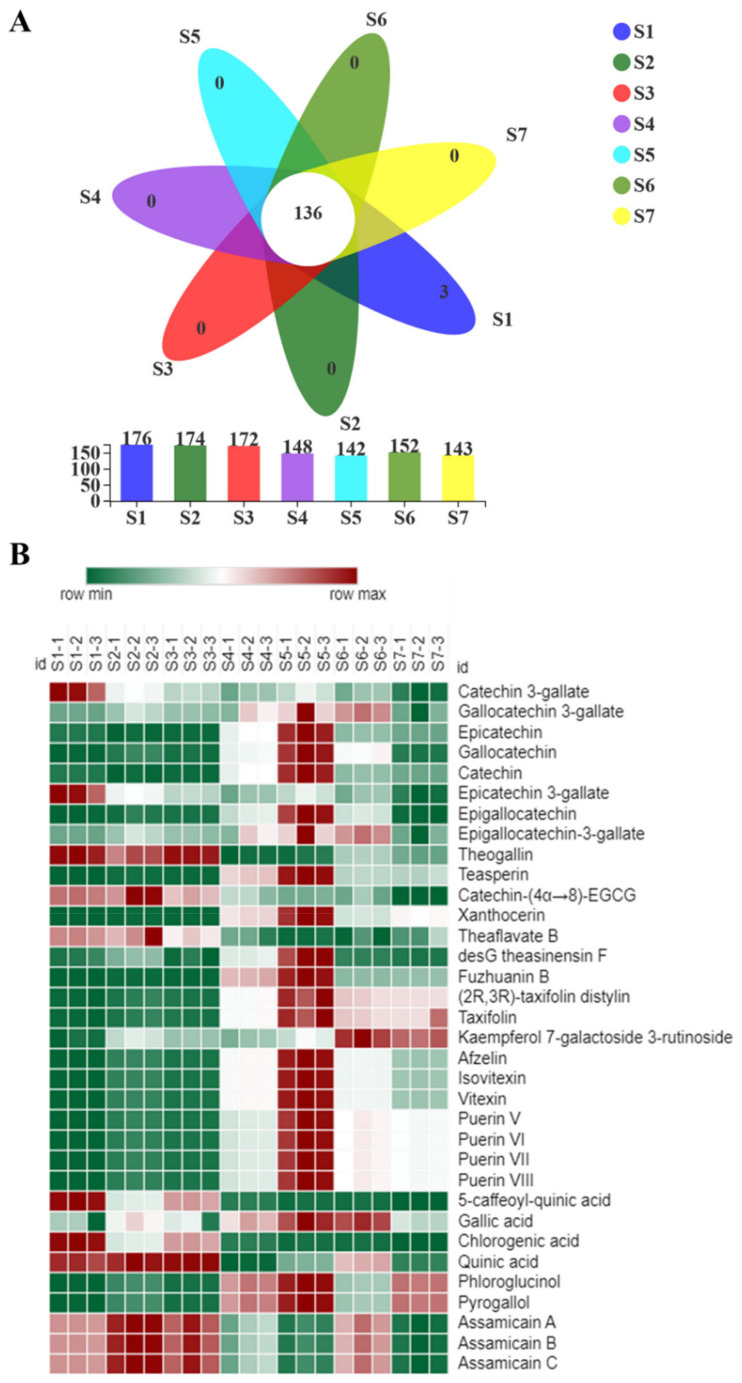
Metabolomics analysis of Fu brick tea samples during processing and storage. (**A**) Venn diagram plot. Numbers represent the identified metabolites. (**B**) Heat map analysis of critical metabolites in different samples. Each column represents a tea sample, and each row represents a critical metabolite. A color-coded scale grading from green to red corresponds to the content of critical metabolite shifting from low to high.

To explore the metabolic changes during FBT processing, LC–MS-based metabolomics coupled with multivariate analysis was applied to identify critical metabolites responsible for metabolite variation caused by FBT processing. Metabolites in tea samples were identified according to authentic standards and tea metabolomics databases. A total of 178 metabolites were identified in 7 samples, including catechins, flavonoids and flavone glycosides, simple phenolics, alkaloids, amino acids, and other metabolites (Table S3). A Venn diagram plot constructed using these data suggested that 136 metabolites were detected in all the tea samples. Among the remaining 42 metabolites, only 3 metabolites were specific to S1 (Figure 4A). Furthermore, the compounds were listed in heat map to provide a more immediate view of the critical metabolites (Figure 4B). These results indicated that FBT processing caused qualitative changes in metabolic profiles. 

**Figure 5:**
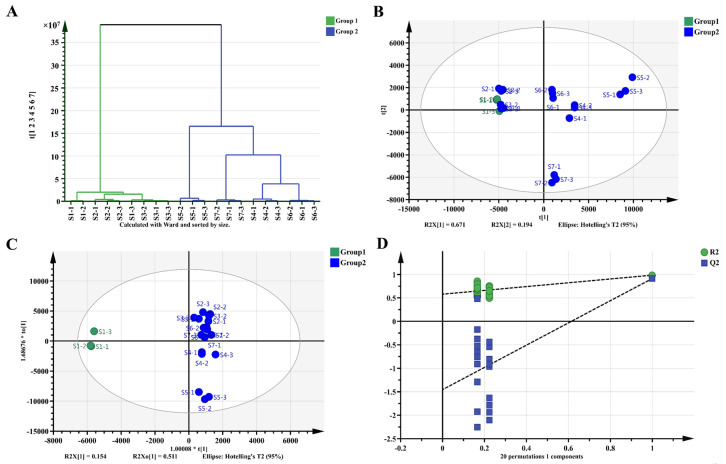
Multivariate analysis of seven FBT samples. (**A**) Dendrogram plot of tea samples. (**B**) PCA score plot, R^2^X = 99.0%, Q^2^ = 96.6%. (**C**) OPLS-DA score plot, R^2^X = 97.1%, R^2^Y = 98.6%, Q^2^ = 91.4%. (**D**) Permutation plot of OPLS-DA, with intercepts of R^2^ =0.579, Q^2^ = −1.46.

A total of 178 metabolites were subjected to multivariate analysis. The SIMCA-P14.1 (14.1.0.2047, Umetrics AB) multivariate statistical software was used to perform PCA and OPLS-DA in order to obtain a general overview of the variance of metabolites in the study. R2 indicates the goodness of the model’s fit and Q2 represents the predictive ability of the model. The dendrogram plot classified the seven samples into two types, where S1 was dramatically different from S2–S7 (Figure 5A). The S1 was visually different from the other sample, as well as the PCA and OPLS-DA (Figure 5B,C) analysis also showed a similar clustering. The OPLS-DA comparison between the S1 and the other seven groups suggested an obvious metabolic differences between the classes in each component. The OPLS-DA models were well constructed, with high R^2^ (0.579) and Q^2^ (−1.46) values representing excellent fit and satisfactory predictive power. (Figure 5D). These results indicated that the fermentation processes changed the metabolic profile of FBT compared to the starting raw material. Considering the concurrent change in the microbiome, these results revealed microbiome changes were a critical factor in the changing metabolic profile. 

Furthermore, based on OPLS-DA with good pairwise discriminations and the cutoff VIP value (VIP > 1.0), a total of 34 critical metabolites were selected to visualize the dynamic changes during FBT processing (Table 3). The content variation of the critical metabolites between samples was represented by the depth of the colors shown in Figure 4B. The results suggested that the content of critical metabolites was high in the flowering period with some exceptions, including catechin, theaflavat B, kaempferol 7-galactoside 3-rutinoside, 5-caffeoyl-quinic acid, and others. Compared with the raw material tea, most critical metabolites in FBT increased significantly during the manufacturing processes.

### 2.6. Measurements of Critical Functional Components of Fu Brick Tea 

The HPLC–MS approach largely focuses on the identification of small molecular endogenous metabolites in organisms. However, the nutritional and healthy ingredients in Fu brick tea are mostly macromolecules, such as polysaccharides, tea polyphenols and catechin. To further confirm critical functional components of Fu brick tea, biochemical methods were used to measure these contents of components. 

#### 2.6.1. Amino Acids

**Figure 6:**
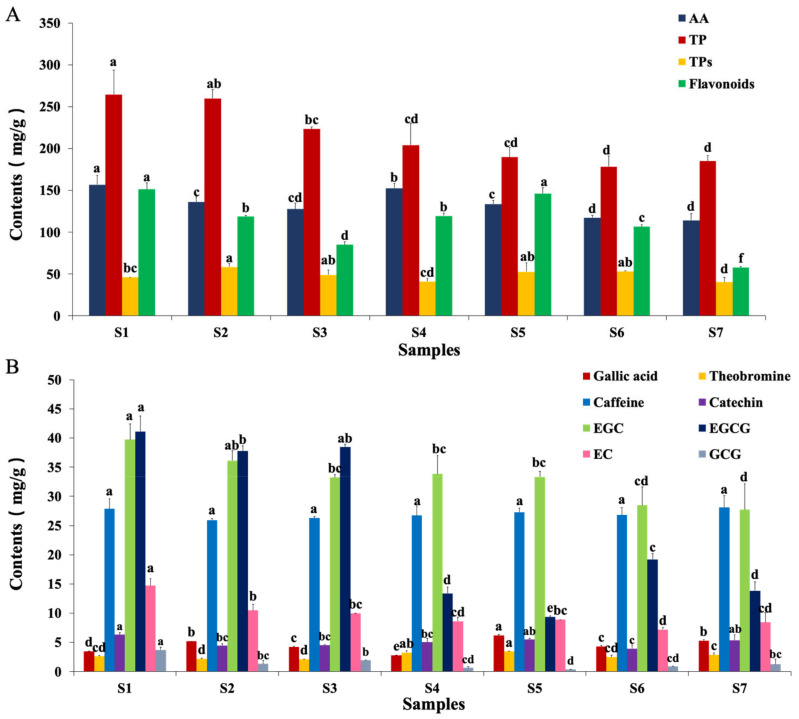
Biochemical measurements of functional components of Fu brick tea. (**A**) Changes of total flavonoids, tea polyphenols (TP), tea polysaccharide (TPs) and free amino acid (AA). (**B**) The contents of Gallic acid, theobromine, caffeine, catechin, catechin, epigallocatechin (EGC), epigallocatechin gallate (EGCG), epicatechin (EC) and galloca-techin gallate (GCG) were determined during processing and storage in Fu brick tea; the data in three replicas, (a–f) expressed *p* < 0.05.

Amino acids are important components that affect the tea taste. Additionally, γ-aminobutyric acid, a nonprotein amino acid, acts as a major inhibitory neurotransmitter in microbial fermentation [19]. HPLC determined 19 amino acids in FBT samples, as shown in Table S4. Most amino acids were reduced during the manufacturing processes, or even disappeared during the ripened period, except for Tyr and Arg. This may have been caused by theanine degradation into Glu and acetylamine, which was produced in hot/humid conditions and by microbial catalysis. Compared with S1, Tyr and Arg were increased 42% and 59.8%, respectively, in S4. The dynamic shift of free amino acids was correlated with the consumption of microbes, as well as the hydrolysis of extracellular enzymes (Figure 6A).

#### 2.6.2. Polyphenol

The health-promoting effect of FBT might be a combined effect of all of the ingredients it contains, especially the polyphenols. Polyphenols in S1 sharply decreased to 178.45 mg/g by the time of the S6 samples (Figure 6A). These unexpected results might have been caused by oxidation from microorganisms present in the FBT sample. Tea polyphenols were decomposed into chatein, chalignin, and phenolic acid. On the other hand, they were oxidized into polyphenol complexes. Catechins account for 60−80% of tea polyphenols, and are the main components that contributes to the antioxidant activity of tea. A previous study reported that catechins had strong antioxidant capacity in vivo or in vitro. [20] Furthermore, the dynamic changes of catechin and gallocatechin were marked by an overall decline (Figure 6B). During dark tea processing, polymeric catechins and gallyolated catechins in tea leaves were gradually hydrolyzed into nongallyolated catechins and gallic acid, which were further degraded into phenolic acids [21,22].

It was reported that galloylated catechins are hydrolyzed during microbial fermentation, with a resultant increase in their hydrolytic products [23]. Meanwhile, nongalloylated catechins are further oxidized and polymerized into tea pigments or degraded into phenolic acids, resulting in their decrease [21]. For this reason, the contents of EC were decreased during fermentation processing. Gallic acid was derived from the hydrolysis of procyanidins and gallyolated catechins, then degraded into methoxyphenolic compounds, such as 1,2-diOMe-benzene, 1,2,3-triOMe-benzene, and 1,2,4-triOMe-benzene during dark tea processing [24]. 

#### 2.6.3. Tea Polysaccharides

Tea polysaccharides are one of the main compounds that contribute to tea flavors, and can relieve the bitter taste from polyphenols, theobromine, chalignin, and other ingredients. The contents of tea polysaccharides remained stable during the manufacturing processes (Figure 6A). This result was mainly due to the tea polysaccharides producing a series of carboxylation reactions, such as intramolecular dehydration, condensation, and polymerization to produce a dark-brown soup color and caramel aroma in high temperature and high humidity. TPs increased in the S2 period, following a decrease in fermentation due to the large reproduction of fungi in the early fermentation period.

#### 2.6.4. Flavonoids 

The total contents of flavonoids were markedly reduced as a result of FBT processing (Figure 6A). This may have been caused by the hydrolysis of flavonoids and flavone glycosides [23]. The flavonoids and their derivatives may have been converted into microbial metabolites during microbial fermentation [25]. A previous study found that flavan-3-ols and procyanidin B3 contributed to the bitter and astringent tastes of tea infusion [26]. The reduction of flavonoids and flavone glycosides may improve the bitter and astringent tastes of FBT. 

#### 2.6.5. Other Notable Changes

The contents of gallic acid, theobromine, EGC, catechin, caffeine, EGCG, EC, GCG, and ECG were investigated to compare the dynamic changes in FBT samples. The results shown in Figure 6B suggested that the contents of EGC, EC, EGCG, and GCG were significantly decreased, and the extent of the decline was dependent on being a complex of catechin. Theobromine and caffeine had no significant difference between S1 and other samples.

Compared with metabolomics changes (Figure 4B), the trends of biochemical changes of critical functional components of FBT including polyphenol, flavonoids, gallic acid, theobromine, EGC, catechin, caffeine, EGCG, EC, GCG and ECG were consistent, proving the reliability of metabolomics methods from another aspect.

### 2.7. Correlation between CRITICAL Metabolites and Microorganisms

**Figure 7:**
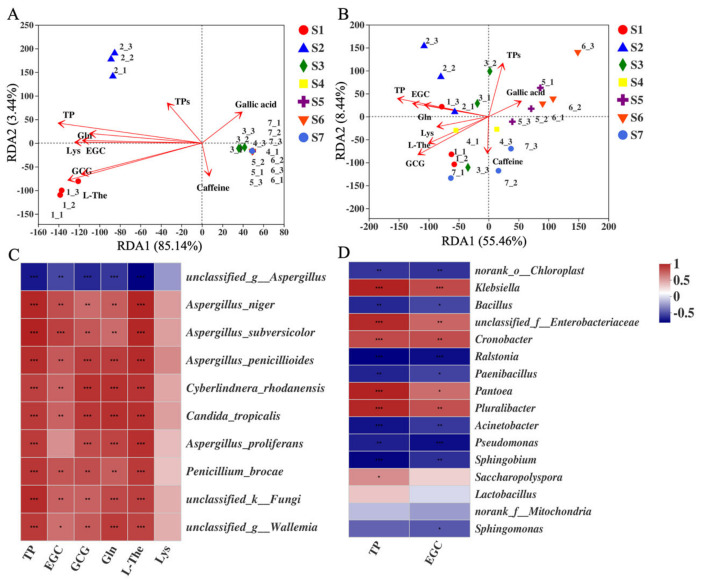
Correlations between microorganisms and metabolites during the processing and storage of Fu brick tea. (**A**) Redundancy analysis (RDA) shows the correlation between the 9 critical metabolites and seven FBT samples, based on fungal OTUs, and (**B**) bacterial OTUs. (**C**) Heat map analysis the correlation between critical metabolites with important fungal species and (**D**) bacteria genus. (note: * 0.01 < *p* ≤ 0.05, ** 0.001 < *p* ≤ 0.01, *** *p* ≤ 0.001).

Multivariate analysis was used to analyze correlation between the critical metabolites and microorganisms during the production of FBT. Based on variance inflation factor (VIF < 10) analysis, nine critical metabolites were screened for redundancy analysis (RDA). The detailed VIF values are shown in Table S5. Among nine critical metabolites, L-The and EGC showed positive correlations with fungal communities in the S1 sample. Gln, TP, and TPs exhibited positive correlations with fungal communities in the S2 sample. Caffeine exhibited positive correlations with fungal communities in the S3–S5 samples. Gallic acid showed negative correlation with all the fungal communities (Figure 7A). The nine critical metabolites were positively correlated with bacterial communities (Figure 7B). TP, EGC, and Gln were positively correlated in the S2 sample. The dynamic changes of L-The, GCG, Lys, and caffeine positively correlated with bacterial communities in the S1 sample, and gallic acid exhibited positive correlations with bacterial communities in S5 sample.

The critical metabolites (*p* ≤ 0.05, R^2^ ≥ 0.4) were analyzed for the top 10 fungi and top 16 bacteria (Figure 7C,D). Except *unclassified_g_Aspergillus*, all the fungi showed positive correlations with six critical metabolites. In particular, L-The and TP displayed a strong correlation with nine species of fungi. As shown in Figure 7C, *Aspergillus_penicillioides* exhibited positive correlations with L-The and TP (R^2^ > 0.8, *p* ≤ 0.001). *Cyberlindnera_rhodanensis* showed positive correlations with L-The, TP, GCG, and Gln (0.5 < R^2^ ≤ 0.8). *Aspergillus_niger* exhibited positive correlations with L-The and TP (*p* ≤ 0.001), and *Aspergillus_proliferans* showed positive correlations with L-The, TP, GCG, and Gln (0.5 < R^2^ ≤ 0.8). The detailed value of correlation coefficients between important fungal species and critical metabolites is exhibited in Figure 7C. As shown in Figure 7D, 5 bacteria (*Cronobacter*, *Klebsiella*, *Pantoea*, *Pluralibacter,* and *unclassified_ f_Enterobacteriaceae)* showed positive correlations with EGC and TP, while the other 11 bacteria correlated negatively with all the critical metabolites. *Cronobacter* exhibited positive correlations with EGC and TP (*p* ≤ 0.001), and *Aspergillus_proliferans* showed positive correlations with L-The, TP, GCG, and Gln (0.5 < R^2^ ≤ 0.8, *p* < 0.01). *Klebsiella, Pantoea*, *Pluraliba,* and *unclassified_ Enterobacteriaceae* showed positive correlations with TP (R^2^ > 0.8) and EGC. In summary, TP had the highest correlation with bacteria, and the dynamic change in bacterial communities was closely related to the decrease of TP.

## 3. Discussion 

Fungi and bacteria are critical factors in the FBT fermentation and ripened steps. In our study, we revealed the fungal and bacterial community modification and metabolite changes during the manufacturing processes of FBT by high-throughput Illumina MiSeq sequencing combined with qPCR, HPLC–MS analysis combined with biochemical measurements, and multivariate analysis. The results, from dynamic changes in the microbial community structure and metabolites, as well as the correlation analysis, indicated strong relationships between metabolites and microorganism communities during the manufacturing processes of FBT.

Fungal community analysis showed *Aspergillus* was dominant in the FBT manufacturing processes. Many species of *Aspergillus* are used in biotechnology for the production of various metabolites, such as antibiotics, organic acids, medicines, or enzymes, or as agents in many food fermentations [27]. *Aspergillus-penicillioid* was the dominate species in primary dark tea, but was quickly replaced by *unclassified_g_Aspergillus* in the middle stage of fermentation. Based on the culture and observation, we surmised this increase of *unclassified_g_Aspergillus* was caused by *Eurotium cristatum*. Many previous reports also showed that *Aspergillus cristatum* (*Eurotium cristatum*) was the dominant fungus during the manufacturing processes of FBT [3,7]. The fungal genus *Eurotium*, which is the teleomorph of *Aspergillus*, has proven to be a rich source of novel bioactive metabolites [28,29]. The richness of *Cyberlindnera_rhodanensis, Aspergillus_niger, Candida tropicali, unclassified_g_Wallemia, Aspergillus subversicol,* and *Aspergillus prolife* were decreased during the FBT manufacturing processes. The richness of *Klebsiella* was the highest among bacterial genera in the S1 sample. The richness of *Cyanobacteria*, *unclassified_f_ Enterobacteriaceae*, *Cronoba, Saccharopolyspora*, *Sphingobium,* and *Enhydrobacter* decreased during the manufacturing processes of FBT. Abundant *Bacillus* and *Thermocatinomyces* were identified in flowering period. In addition, *Lactobacillus* was the dominate bacteria in the ripened samples.

In the exploring of the dynamic evolution of microbial during the manufacturing processes and storage of Fu brick tea, we found many unclassified new microbes, including fungi and bacteria. This is a very interesting discovery, in that these strains only grow in the media of Fu brick tea components. The isolation and characterization of unclassified microbes from FBT is a new field to develop FBT functions more deeply.

Considering the lack of a systematic comparative study of different FBT processes samples, we carried out a comprehensive multivariate analysis focusing on microbial community structure shift, correlation between microbiomes, and metabolites during FBT processing. The multivariate analysis results showed that dynamic evolution of fungal community structure samples could be separated into three periods: designated stage I (S1), stage II (S2), and stage III (S3–S7). Moreover, the dynamic evolution of bacterial community structure samples could be separated into two periods, designated stage I (S1–S6) and stage II (S7). These results indicated that the bacterial community structure was more stable compared to the fungal community structure during the fermentation periods. 

Based on LC–MS metabolomics and multivariate analysis, 178 metabolites were identified, and 34 critical metabolites were selected for metabolic variation among the samples. The result indicated that the critical metabolites were significantly different between primary dark tea and later-manufactured tea samples. Moreover, piling-fermentation and flowering fermentation had a varied effect on metabolites. Meanwhile, our metabolic analysis showed that polyphenols were significantly decreased. These changes probably resulted from moisture and heat reactions and microbial metabolism. The increase of gallic acid may have been related to the hydrolysis of galloylated catechins (polyphenols) and microbial metabolism, which might be helpful in some bioactivity of FBT [8]. The content of flavonoids showed a significant increase in the flowering fermentation and ripened periods. At the end of the flowering and ripened periods, the flavonoids began to decrease, which not only reduced their bioactivities, but also reduced the bitter taste of FBT. Furthermore, new metabolites such as three triterpenoids [30], one norisoprenoid [31], and new B-ring fission catechin (flavan-3-ols) derivatives [32,33,34] were determined in FBT. Notably, these B-ring fission catechins have not been found previously in other tea products, indicating the biochemical profiles of FBT were influenced uniquely by the fermentation processes. Moreover, the effect of bacteria on metabolites was stronger than that of fungi, which may have played an auxiliary role in substance transformation or could have produced oxidases, which accelerated the transformation reaction and reduced the fermentation cycle.

Different dominant species in different periods determined the compositions of critical functional components of Fu brick tea. *Eurotium cristatum*, as the most dominant fungal species in each period, secrets amylase and oxidase, which catalyze the conversions of starch into monosaccharides and the oxidation of polyphenol compounds into substances beneficial to the human body, thereby improving and optimizing the taste and other characteristics of tea [7,8]. *Aspergillus_niger* dominated in S1 and disappeared in late periods, and produces a large number of intracellular and extracellular enzymes, which can effectively convert almost all macromolecular compounds, such as polyphenols, protein, flavonoids, pigments, theanine, and tea polysaccharides in the postfermentation period [4,5]. *Cyanobacteria*, as a dominant bacterial species in all periods, was involved in the metabolisms of macromolecular compounds such as tea polysaccharides, flavonoids, theanine, and tea polyphenols. *Bacillus* was abundant in S5 and S7, and generated some oxidases related to the production of gallic acid and theobromine. The change trends of catechins and according derivatives, flavonoids, and flavonoid glycosides and simple phenols were consistent with different dominant species in different periods.

In this study, we revealed the correlation between the microbiome and metabolomics during the processing and storage of Fu brick tea. Based on this discovery, it is rational to improve compositions of bioactive compounds of FBT through exogenous inoculation of specific microorganisms to produce specific flavor FBT and improve the quality of FBT. Moreover, it is necessary to isolate and characterize unclassified microbes from FBT for further developing the value of FBT.

## 4. Experimental Section 

### 4.1. Chemicals and Materials

All standard reagents involved in the experiments were purchased from Yuanye Biotechnology Company (Shanghai, China). The LC–MS grade acetonitrile, formic acid, and methanol were purchased from Sigma Aldrich (St. Louis, MO, USA). Other reagents were analytical grade from China National Medicines Corporation Ltd (Beijing, China).

FBT processes samples were obtained from Hunan Zhongcha Tea Industry Group (China). Generally, the primary dark tea (S1) used as raw material was moistened with water and piled as windrows in the fermentation room. During the pile-fermentation, the fermented tea was artificially turned over, and this processes was stopped when the fermented tea mass was reddish-brown and free from the astringent taste. After pile-fermentation, teas were collected and pressed, after which some of the special fungi formed spores. We named this period “flowering”. In total, 7 samples were collected and labeled as primary dark tea (S1), middle stage of piling-fermentation of tea materials (S2), later stage of piling-fermentation of tea materials (S3), flowering 6 days (S4), flowering 9 days (S5), ripened 180 days (S6), and ripened 540 days (S7).

### 4.2. DNA Extraction, Polymerase Chain Reaction Amplification, and Sequence 

Microbial DNA was extracted from the samples using a FastDNA^®^ Spin Kit (MP Biomedicals, Norcross, GA, USA) as per manufacturer’s instructions. The V3-V4 (338-806) region of the bacterial 16S rRNA gene was amplified from the extracted DNA using forward primer 338F (5′-ACTCCTACGG GAGGCAGCA-3′) [35] and reverse primer 806R (5′-GGACTACHVGGGTWTCTAAT-3′) [36]. The ITS (300-700) region of the fungal internal transcribed spacer was amplified from the extracted DNA using forward primer ITS1F (5’-CTTGGTCATTTAGAGGAAGTAA-3’) and reverse primer ITS2R (5’-GCTGCGTTCTTCATC GATGC-3’). The quality of DNA samples was evaluated by electrophoresis on 2% agarose gels. Each DNA sample was amplified in triplicate PCR reactions with a total volume of 20 µL. The PCR products were purified with an AxyPrep DNA Gel Extraction Kit (Axygen Biosciences, Union City, CA, USA). Purified PCR products were quantified using a QuantiFluotTM DNA assay Kit (Promega, Madison, WI, USA). Following quantification, the mixed sample amplicon sequencing was performed using the Illumina Miseq PE300 /NovaSeq PE250 system by Majorbio Bio-Pharm Technology Co., Shanghai, China. 

### 4.3. Sequence Processing and Analysis 

The raw sequencing reads were demultiplexed using Trimmomatic (v1.7.0) [37], FLASH (version 1.2.7 http://www.cbcb.umd.edu/software/flash accessed on 10 October 2021) [38], UPARSE (version 7.1 http://drive5.com/uparse/ accessed on 10 October 2021) [39], and UCHIME (verson 4.2 http://drive5.com/uchime accessed on 10 October 2021) [40] software for denoising, trimming, quality filtering, pairing, and aligning. The resulting sequences were clustered into operational taxonomic units (OTUs) at 97% similarity using UPARSE software. Then, the representative sequence of each OTU was assigned to taxonomic information using the ribosomal database project (RDP) classifier (http://rdp.cme.msu.edu/ accessed on 10 October 2021) with a 70% threshold by comparing with the Silva database (SSU128). The community richness index (CHAO index), community diversity (Shannon index), and the Good’s coverage of sequencing were estimated using the mothur software (version 1.30 http://www.mothur.org accessed on 10 October 2021). 

### 4.4. Standard Curve Generation and Quantitative PCR (qPCR) Detection of Fungi and Bacteria during Processing and Storage of Fu Brick Tea

The 188 bp fragment (091-279) of yeast (BY4741, Saccharomyces cerevisiae) ITS gene obtained by PCR with specific primers (F: 5’- CGCGGATCCCCAGCCGGGCCTGCGCT TAAG, R: 5’- CCGCTCGAGCCT CTGGGCCCCGATTGCTCG) was inserted into Bam HI and Xho I sites in the p416-TEF vector for TEF2 gene-promoter-mediated constitutive expression in yeast. This p416-TEF-ITS (188) plasmid was used for fungal standard curve generation. The same method was applied for bacterial standard curve generation. The 659 bp fragment (031-690) of *E. coli* 16s rDNA obtained by PCR with specific primers (F: 5’-CGCGGATCCCGGCAGGCCTAACACATGCAAG, R: 5’- CCGCTCGA GGCATTTCACCGCTACACCTG) was inserted into the Bam HI and Xho I sites in the p416-TEF vector. All plasmids were confirmed by sequencing. The cycle threshold (Ct) value, obtained from 10-fold serial dilutions of plasmid DNA in deionized water to produce 5 different concentrations, was used to prepare standard curves for quantitative PCR (qTOWER, 3.0G) using primer sets that were specific for fungi (F: 5′- CCAGCCGGGCC TGCGCTTAAG, R: 5′- CCTCTGGGCCCCGATTG CTCG) and bacteria (F: 5′- ACTCCTACGGGAGGCAGCAG, R: 5′- ATTACCGCGGCTGG) [41]. Common molecular biology techniques, including plasmid amplification and purification using Escherichia coli, followed previously established methods [42]. Genomic DNA samples of different processes of Fu brick tea were extracted by DNA extraction kit (E.Z.N.A. ® Soil DNA kit, Omega Bio-tek, Norcross, GA, US), followed by quantitative PCR using the same primer sets as those of the standard curve qPCR.

### 4.5. Sample Preparation 

#### 4.5.1. Determination of Main Active Compounds 

Tea polyphenol (TP) was determined using the iron tartrate colorimetric method [43]. The contents of theobromine, caffeine (CAF), gallic acid (GA), and catechins, including (+)-catechin (C), (−)-epicatechin (EC), (−)-epigallocatechin (EGC), epicatechin gallate (ECG), (−)-Galloca-techin gallate (GCG), and epigallocatechin gallate (EGCG) in the FBT samples were determined by high-performance liquid chromatography (HPLC) using a Waters 2695 HPLC System (Waters, Milford, MA, USA). Total flavonoid content was determined using the colorimetric method (SZDB/Z 349-2019) with rutin as the standard. The qualitative analysis of tea pigment (theabrownins) was carried out using the method of Wang et al [44]. A 0.1 g tea sample was extracted with 3 mL methanol (70% v/v) for 20 min in a 60 °C water bath. The extraction was repeated 2 times. After cooling, thesolution was passed through a 0.2 μm syringe filter and used for HPLC analysis. The detection wavelength was 280 nm.

#### 4.5.2. Determination of Tea Free Amino Acids

Total amino acids (AAs) of the tea samples were quantified using the ninhydrin method [45]. A 0.15 g tea powder sample was placed in a test tube and extracted with 10 mL hot water for 20 min in a 95 °C water bath. The extraction was repeated 2 times, after centrifugation (5000 rpm, 5 min), then supernatants were combined and diluted to 25 mL. Before analysis, the solution was filtered with a 0.2 μm syringe filter. The detection wavelength was 570 nm. 

#### 4.5.3. Determination of Tea Polysaccharides (TP_S_)

The anthrone method was used to determine the water-soluble sugar in tea samples (SN/T 4260-2015) with glucose as the standard. A 0.2 g tea powder sample was extracted with 20 mL hot water for 60 min in a 95 °C water bath. To keep the tea powder suspension in the extractant, tubes were vibrated every 10 min. The obtained supernatant was diluted to 50 mL and mixed with anthrone solution. The detection wavelength was 620 nm. 

### 4.6. HPLC–MS Analysis 

FBT samples were determined by high-performance liquid chromatography (HPLC) using a Waters GRX-9123A HPLC system. The separation was carried out using a BEH C18 column (1.7 μm, 2.1 mm × 100 mm, Waters, USA) and the column was maintained at 45 °C and eluted with a gradient of 1–100% methanol containing 0.1% (*v*/*v*) formic acid over 15 min at a flow rate of 0.4 mL/min. A 1 μL sample was injected into the column, and the gradient elution condition followed a previous publication [18]. 

The mass spectrometer was operated in negative ionization mode over a full scan range of 50−1000 *m*/*z* with the following settings: gas temperature 450 °C, gas flow 15 L/min, source temperature 115 °C, capillary voltage 2 kV, cone voltage 40 V, and collision energy 6 eV. The internal standard (DL-4-chlorophenylalanine) was applied to partially compensate for the concentration bias of a given metabolite among the tea samples.

### 4.7. Multivariate Analysis

The raw data from LC–MS were converted to acquire format using MataboAnalyst software (https://www.metaboanalyst.ca/ accessed on 10 October 2021). Hierarchical cluster analysis (HCA), unsupervised principal component analysis (PCA), analysis of variance (ANOVA) and supervised orthonormal partial least-squares discriminant analysis (OPLS-DA) were performed using SIMCA-P +software (version 14.1, Umetrics AB, Sweden). The HCA visualized the relationship of tea samples by simultaneously plotting the respective dendrograms and their connections. The PCA examined the intrinsic variation in the collected data matrix and distinguished the differences among these FBT processing samples, with OPLS-DA applied to classify samples of solely Y variables. The critical metabolites responsible for metabolomics variation caused by FBT processing were identified using OPLS-DA modeling combined with a threshold of variable importance project (VIP) > 1.0. The heat map analysis was carried out using the website https://software.broadinstitute.org/morpheus/ (accessed on 10 October 2021).

All experiments were carried out in triplicate, and the results were expressed as mean values followed by the standard deviation (n = 3). One-way ANOVA with Dunnett’s multiple comparisons test was used to distinguish the significance level of metabolites between groups. The linear correlation between the inhibitory effects of digestive enzymes and the chemical composition of the samples was analyzed by SMICA-P software, and the probability values were calculated; p-values below 0.05 were considered significant. 

## 5. Conclusions 

Our study systematically described the dynamic evolution of the microbiome and metabolites during the FBT manufacturing processes, and confirmed the influence of fungi and bacteria on critical metabolites. The results demonstrated that an LC–MS approach combined with multivariate analysis was able to collect sufficient information of metabolic profiles to consequently provide a correlation between critical metabolites and microorganisms, and revealed a possible way to control FBT quality by adding specific microbial species to increase metabolic benefits. Additionally, these findings advanced our understanding of the changes to the fungal and bacterial communities during manufacturing, and help to elucidate the potential functions of specific genera in the development of the special characteristics of FBT.

## Figures and Tables

**Table 1:** The optimized sequence information obtained via fungal high-throughput sequencing.

Amplified Region	Sample	Sequences	Bases (bp)	Average Length
ITS1F_ITS2R	21	13,780,204	312,864,617	226.9

**Table 2:** The optimized sequence information obtained via bacterial high-throughput sequencing.

Amplified Region	Sample	Sequences	Bases (bp)	Average Length
338F_806R	21	1,055,795	439,213,816	416.2

**Table 3:** Critical metabolites (VIP > 1.0) responsible for the metabolomics variations caused by FBT processing.

No.	Rt (min)	*m*/*z*	Formula	Mass Error (mDa)	VIP	Tentative Identification
1	4.07	305.0664	C_15_H_14_O_7_	−0.3	3.63871	Gallocatechin
2	4.7	289.0712	C_15_H_14_O_6_	−0.5	3.33493	Epicatechin
3	4.7	289.0712	C_15_H_14_O_6_	−0.5	3.33493	Catechin
4	5.85	441.0825	C_22_H_18_O_10_	−0.2	2.87242	Catechin 3-gallate
5	5.85	441.0825	C_22_H_18_O_10_	−0.2	2.87242	Epicatechin 3-gallate
6	4.74	457.0775	C_22_H_18_O_11_	−0.2	2.16752	Gallocatechin 3-gallate
7	4.74	457.0775	C_22_H_18_O_11_	−0.2	2.16752	Epigallocatechin-3-gallate
8	4.39	311.0768	C_13_H_14_O_6_	−0.4	2.06244	Fuzhuanin B
9	4.75	915.1625	C_44_H_36_O_22_	0	1.94329	Assamicain A
10	4.75	915.1625	C_44_H_36_O_22_	0	1.94329	Assamicain B
11	4.75	915.1625	C_44_H_36_O_22_	0	1.94329	Assamicain C
12	3.78	305.0666	C_15_H_14_O_7_	−0.1	1.91098	Epigallocatechin
13	4.49	349.058	C_15_H_12_O_7_	1.5	1.82695	Taxifolin
14	4.49	349.058	C_15_H_12_O_7_	1.5	1.81141	(2R,3R)-taxifolin distylin
15	6.08	755.2041	C_33_H_40_O_20_	0.1	1.80491	Kaempferol 7-galactoside 3-rutinoside
16	2.65	125.0241	C_6_H_6_O_3_	−0.3	1.74387	Phloroglucinol
17	2.65	125.0241	C_6_H_6_O_3_	−0.3	1.74387	Pyrogallol
18	3.46	343.0668	C_14_H_16_O_10_	−0.3	1.67692	Theogallin
19	4.3	353.0874	C_16_H_18_O_9_	−0.4	1.57482	5-caffeoyl-quinic acid
20	4.3	353.0874	C_16_H_18_O_9_	−0.4	1.57482	Chlorogenic acid
21	0.8	191.0556	C_7_H_12_O_6_	−0.5	1.52054	Quinic acid
22	4.75	169.0137	C_7_H_6_O_5_	−0.6	1.50629	Gallic acid
23	6.31	231.0656	C_13_H_12_O_4_	−0.7	1.49003	Teasperin
24	5.92	431.0983	C_21_H_20_O_10_	−0.1	1.35541	Afzelin
25	5.92	431.0983	C_21_H_20_O_10_	−0.1	1.35541	Isovitexin
26	5.92	431.0983	C_21_H_20_O_10_	−0.1	1.35541	Vitexin
27	5.94	293.0663	C_13_H_12_O_5_	−0.3	1.2387	Xanthocerin
28	4.49	745.1406	C_37_H_30_O_17_	−0.4	1.06786	Catechin-(4α→8)-EGCG
29	4.67	416.1347	C_21_H_23_NO_8_	−0.4	1.03255	Puerin V
30	4.67	416.1347	C_21_H_23_NO_8_	−0.4	1.03255	Puerin VI
31	4.67	416.1347	C_21_H_23_NO_8_	−0.4	1.03255	Puerin VII
32	4.67	416.1347	C_21_H_23_NO_8_	−0.4	1.03255	Puerin VIII
33	4.05	593.1302	C_30_H_26_O_13_	0.2	1.03186	desG theasinensin F
34	4.49	745.1406	C_36_H_28_O_15_	−0.5	1.0057	Theaflavate B

Note: “0” suggests that the given metabolite vanished after microbial fermentation or FBT processing.

## Data Availability

Data is contained within the article or supplementary materials.

## Supplementary Materials

The following are available online at https://www.mdpi.com/article/10.3390/metabo11100703/s1, Figure S1: Rarefaction curve analysis of the fungal community in the fermentation process of FBT. Fungus sobs curve (A); fungus Shannon–Wiener curve (B), Figure S2: The number of operational taxonomic units (OTUs) and alpha diversity estimators for fungal communities in the FBT samples, Figure S3: Rarefaction curve analysis of the bacterial community in the fermentation process of FBT. Bacterial sobs curve (A); bacterial Shannon-Wiener curve (B), Figure S4: The number of operational taxonomic units (OTUs) and alpha diversity estimators for bacterial communities in the FBT samples, Table S1: Diversity analysis of fungal communities in the FBT samples, Table S2: Diversity analysis of bacterial communities in the FBT samples, Table S3: Total of 178 metabolites identified in FBT samples, Table S4: HPLC detection of 19 amino acids in the FBT samples (mg/g), Table S5. VIF value of nine critical ingredients.
