# Supplementary material for: Dynamic Evolution and Correlation between Metabolites and Microorganisms during Manufacturing Process and Storage of Fu Brick Tea
Source: Metabolites. 2021 Oct 13;11(10):703. doi: 10.3390/metabo11100703 (PMC8539874; doi:10.3390/metabo11100703)
Supplement: Supplementary file 1 [file metabolites-11-00703-s001.zip › metabolites-1398174-Supp-final.pdf]

Supplementary Table S1. Diversity analysis of fungal communities in FBT samples

Sample	Community diversity index		Community richness index		Coverage%
	Simpson	Shannon	Ace	Chao1	
S1	0.23±0.01c	1.85±0.03b	126.20±6.03a	127.29±10.73a	99
S2	0.19±0d	2.08±0.01a	96.00±7.24a	89.76±4.19b	99
S3	0.88±0.02b	0.37±0.07c	96.20±23.96a	79.06±6.85b	99
S4	0.99±0a	0.03±0.003d	34.99±20.60bc	18.50±7.78cd	99
S5	1.00±0.001a	0.01±0.002d	7.33±6.66c	7.00±1.32d	99
S6	1.00±0.001a	0.02±0.002d	14.93±9.31bc	11.75±6.63d	99
S7	0.99±0.001a	0.03±0.003d	44.95±22.71b	44.17±30.58c	99

Supplementary Table S2. Diversity analysis of bacterial communities in FBT samples

Sample	Community diversity index		Community richness index		Coverage%
	Simpson	Shannon	Ace	Chao1	
S1	0.22±0.02d	1.84±0.07c	253.20±15.44b	187.52±15.30e	99
S2	0.24±0.01cd	1.82±0.02c	255.40±18.03b	193.08±26.60d	99
S3	0.31±0.003c	1.70±0.05cd	258.92±71.43b	223.56±55.66cd	99
S4	0.21±0.10d	2.58±0.32b	295.67±28.12ab	294.50±25.85ab	99
S5	0.54±0.04b	1.38±0.08d	272.07±20.30b	272.29±19.95bc	99
S6	0.73±0.01a	0.87±0.04e	251.65±17.52b	250.90±16.54bc	99
S7	0.14±0.07e	3.41±0.31a	341.65±8.85a	342.09±8.57a	99

Supplementary Table S3. Total of 178 metabolites identified in FBT samples

no.	RT (min)	m/z	Formula	Mass error (mDa)	tentative identification
1	5.85	441.0825	$C_{22}H_{18}O_{10}$	-0.2	Catechin 3-gallate
2	4.74	457.0775	$C_{22}H_{18}O_{11}$	-0.2	Gallocatechin 3-gallate
3	5.37	273.0762	$C_{15}H_{14}O_5$	-0.7	EA
4	4.4	425.0874	$C_{22}H_{18}O_9$	-0.4	EA 3-gallate
5	4.7	289.0712	$C_{15}H_{14}O_6$	-0.5	Epicatechin
6	6.75	357.1338	$C_{20}H_{22}O_6$	-0.6	pinoresinol
7	4.07	305.0664	$C_{15}H_{14}O_7$	-0.3	Gallocatechin
8	4.49	349.058	$C_{15}H_{12}O_7$	1.5	(2R,3R)-taxifolin distylin
9	4.81	635.0889	$C_{27}H_{24}O_{18}$	-0.1	1,2,6-tri-G-glu
10	4.04	483.0783	$C_{20}H_{20}O_{14}$	0.3	1,2-di-G-glu
11	4.04	483.0783	$C_{20}H_{20}O_{14}$	0.3	1,3-di-G-glu
12	4.81	635.0889	$C_{27}H_{24}O_{18}$	-0.1	1,4,6-tri-G-glu

13	4.04	483.0783	$C_{20}H_{20}O_{14}$	0.3	1,6-di-G-glu
14	2.49	331.0668	$C_{13}H_{16}O_{10}$	-0.3	1-G-glu
15	4.08	167.0341	$C_8H_8O_4$	-0.8	2,5-Dihydroxyphenylacetic acid
16	4.04	483.0783	$C_{20}H_{20}O_{14}$	0.3	2,6-di-G-glu
17	10.62	285.0401	$C_{15}H_{10}O_6$	-0.4	3',4',5,7-tetraOH-flavone
18	4.93	153.019	$C_7H_6O_4$	-0.4	3,5-diOH-benzoic acid
19	4.4	183.0293	$C_7H_6O_3$	-0.6	3-Hydroxybenzoic acid
20	4.81	337.0925	$C_{16}H_{18}O_8$	-0.4	3-coumaroyl-quinic acid
21	3.93	353.0876	$C_{16}H_{18}O_9$	-0.2	4-caffeoyl-quinic acid
22	4.81	337.0925	$C_{16}H_{18}O_8$	-0.4	4-coumaroyl-quinic acid
23	4.3	353.0874	$C_{16}H_{18}O_9$	-0.4	5-caffeoyl-quinic acid
24	4.81	337.0925	$C_{16}H_{18}O_8$	-0.4	5-coumaroyl-quinic acid
25	3.63	241.0825	$C_{10}H_{14}N_2O_5$	-0.5	7-Me-xanthine
26	0.69	333.0589	$C_{16}H_{14}O_8$	-2.7	8-carboxyl-catechin
27	0.81	209.066	$C_8H_{10}N_4O_3$	-2	8-oxocaffeine
28	5.92	431.0983	$C_{21}H_{20}O_{10}$	-0.1	Afzelin
29	2.73	134.0469	$C_3H_7NO_2$	1	Alanine
30	3.98	152.0348	$C_7H_7NO_3$	-0.5	Aminosalicylic Acid
31	0.81	175.0244	$C_6H_8O_6$	-0.4	Ascorbic acid
32	6.8	447.0929	$C_{21}H_{20}O_{11}$	-0.4	Asiaticalin
33	0.68	132.0298	$C_4H_7NO_4$	-0.5	Aspartic acid
34	4.75	915.1625	$C_{44}H_{36}O_{22}$	0	Assamicain A
35	4.75	915.1625	$C_{44}H_{36}O_{22}$	0	Assamicain B
36	4.75	915.1625	$C_{44}H_{36}O_{22}$	0	Assamicain C
37	6.8	447.0929	$C_{21}H_{20}O_{11}$	-0.4	Astragalin
38	6.5	593.1509	$C_{27}H_{30}O_{15}$	-0.3	Biorobin
39	6.35	637.1554	$C_{31}H_{28}O_{12}$	-0.8	Bis(8-epicatechinyl)-methane
40	4.7	289.0712	$C_{15}H_{14}O_6$	-0.5	Catechin
41	9.46	593.1299	$C_{30}H_{26}O_{13}$	-0.2	Catechin-(4α→8)-EGC
42	4.49	745.1406	$C_{37}H_{30}O_{17}$	-0.4	Catechin-(4α→8)-EGCG
43	3.82	109.0293	$C_6H_6O_2$	-0.2	Catechol
44	5.31	415.1605	$C_{19}H_{28}O_{10}$	-0.5	Chakanoside II
17	10.62	285.0401	$C_{15}H_{10}O_6$	-0.4	3',4',5,7-tetraOH-flavone
18	4.93	153.019	$C_7H_6O_4$	-0.4	3,5-diOH-benzoic acid
19	4.4	183.0293	$C_7H_6O_3$	-0.6	3-Hydroxybenzoic acid
20	4.81	337.0925	$C_{16}H_{18}O_8$	-0.4	3-coumaroyl-quinic acid
21	3.93	353.0876	$C_{16}H_{18}O_9$	-0.2	4-caffeoyl-quinic acid
22	4.81	337.0925	$C_{16}H_{18}O_8$	-0.4	4-coumaroyl-quinic acid
23	4.3	353.0874	$C_{16}H_{18}O_9$	-0.4	5-caffeoyl-quinic acid

24	4.81	337.0925	$C_{16}H_{18}O_8$	-0.4	5-coumaroyl-quinic acid
25	3.63	241.0825	$C_{10}H_{14}N_2O_5$	-0.5	7-Me-xanthine
26	0.69	333.0589	$C_{16}H_{14}O_8$	-2.7	8-carboxyl-catechin
27	0.81	209.066	$C_8H_{10}N_4O_3$	-2	8-oxocaffeine
28	5.92	431.0983	$C_{21}H_{20}O_{10}$	-0.1	Afzelin
29	2.73	134.0469	$C_3H_7NO_2$	1	Alanine
30	3.98	152.0348	$C_7H_7NO_3$	-0.5	Aminosalicylic Acid
31	0.81	175.0244	$C_6H_8O_6$	-0.4	Ascorbic acid
32	6.8	447.0929	$C_{21}H_{20}O_{11}$	-0.4	Asiaticalin
33	0.68	132.0298	$C_4H_7NO_4$	-0.5	Aspartic acid
34	4.75	915.1625	$C_{44}H_{36}O_{22}$	0	Assamicain A
35	4.75	915.1625	$C_{44}H_{36}O_{22}$	0	Assamicain B
36	4.75	915.1625	$C_{44}H_{36}O_{22}$	0	Assamicain C
37	6.8	447.0929	$C_{21}H_{20}O_{11}$	-0.4	Astragalin
38	6.5	593.1509	$C_{27}H_{30}O_{15}$	-0.3	Biorobin
39	6.35	637.1554	$C_{31}H_{28}O_{12}$	-0.8	Bis(8-epicatechinyl)-methane
40	4.7	289.0712	$C_{15}H_{14}O_6$	-0.5	Catechin
41	9.46	593.1299	$C_{30}H_{26}O_{13}$	-0.2	Catechin-(4α→8)-EGC
42	4.49	745.1406	$C_{37}H_{30}O_{17}$	-0.4	Catechin-(4α→8)-EGCG
43	3.82	109.0293	$C_6H_6O_2$	-0.2	Catechol
44	5.31	415.1605	$C_{19}H_{28}O_{10}$	-0.5	Chakanoside II
45	4.46	473.0722	$C_{22}H_{18}O_{12}$	-0.3	Chicoric acid
46	4.12	387.1295	$C_{16}H_{22}O_8$	-0.2	Coniferin
47	7.6	193.05	$C_9H_8O_2$	-0.7	Cinnamic acid
48	4.35	145.029	$C_9H_6O_2$	-0.5	Coumarin
49	4.81	337.0925	$C_{16}H_{18}O_8$	-0.4	Coumaroylquinic acid
50	4.05	593.1302	$C_{30}H_{26}O_{13}$	0.2	desG theasinensin F
51	4.26	321.0253	$C_{14}H_{10}O_9$	0.1	Digallic acid
52	4.49	745.1406	$C_{37}H_{30}O_{17}$	-0.4	EC-(4β→8)-EGCG
53	4.92	897.1537	$C_{44}H_{34}O_{21}$	1.7	ECG-(4β→6)-EGCG
54	4.92	897.1537	$C_{44}H_{34}O_{21}$	1.7	ECG-(4β→8)-EGCG
55	4.49	745.1406	$C_{37}H_{30}O_{17}$	-0.4	EGC-(4β→8)-ECG
56	4.92	897.1537	$C_{44}H_{34}O_{21}$	1.7	EGCG-(4β→6)-ECG
57	5.85	881.1578	$C_{44}H_{34}O_{20}$	0.8	EA 3-gallate-(4β→6)-EGCG
58	5.68	471.0929	$C_{23}H_{20}O_{11}$	-0.3	EGCG-4''-Me
59	10.5	269.0451	$C_{15}H_{10}O_5$	-0.4	Emodin
60	7.41	399.0722	$C_{20}H_{16}O_9$	0.1	Epitheaflagallin
61	4.46	473.0722	$C_{21}H_{16}O_{10}$	-0.3	Epitheaflavic acid
62	0.69	333.0589	$C_{15}H_{12}O_6$	-2.7	Eriodictyol

63	3.46	365.0489	$C_{15}H_{12}O_8$	-2.5	Fuzhuanin A
64	4.39	311.0768	$C_{13}H_{14}O_6$	-0.4	Fuzhuanin B
65	4.9	335.0764	$C_{16}H_{16}O_8$	-0.8	Fuzhuanin D
66	4.75	169.0137	$C_7H_6O_5$	-0.6	Gallic acid
67	9.46	593.1299	$C_{30}H_{26}O_{13}$	-0.2	GC-(4α→8)-EC
68	4.4	183.0293	$C_7H_6O_3$	-0.6	Gentisate aldehyde
69	3.82	153.019	$C_7H_6O_4$	-0.3	Gentisic acid
70	9.14	301.035	$C_{15}H_{10}O_7$	-0.4	Herbacetin
71	0.76	135.0296	$C_5H_4N_4O$	-1.7	Hypoxanthine
72	7.6	193.05	$C_{10}H_{10}O_4$	-0.7	Isoferulic acid
73	6.05	463.0877	$C_{21}H_{20}O_{12}$	-0.5	Isoquercitrin
74	5.77	609.1462	$C_{26}H_{28}O_{14}$	0.1	Isoschaftoside
75	3.85	609.1255	$C_{29}H_{24}O_{12}$	0.5	Isotheaflavin
76	4.16	761.1363	$C_{36}H_{28}O_{16}$	0.3	Isotheaflavin 3'-gallate
77	5.92	431.0983	$C_{21}H_{20}O_{10}$	-0.1	Isovitexin
78	6.16	577.1552	$C_{27}H_{30}O_{14}$	-1.1	Kaempferitrin
79	10.62	285.0401	$C_{15}H_{10}O_6$	-0.4	Kaempferol
80	6.5	593.1509	$C_{27}H_{30}O_{15}$	-0.3	Kaempferol 3-Rha-galactoside
81	6.5	593.1509	$C_{27}H_{30}O_{15}$	-0.3	Kaempferol 3-rutinoside
82	6.08	755.2041	$C_{33}H_{40}O_{20}$	0.1	Kaempferol 7-galactoside 3-rutinoside
83	12.86	249.1851	$C_{15}H_{24}$	-1	Longifolene
84	10.62	285.0401	$C_{15}H_{10}O_6$	-0.4	Luteolin
85	0.82	133.014	$C_4H_6O_5$	-0.2	Malic acid
86	6.75	357.1338	$C_{20}H_{22}O_6$	-0.6	Matairesinol
87	4.4	183.0293	$C_8H_8O_5$	-0.6	Me 3,4,5-triOH-benzoate
88	9.14	301.035	$C_{15}H_{10}O_7$	-0.4	Morin
89	7.35	317.0302	$C_{15}H_{10}O_8$	-0.1	Myricetin
90	5.25	479.0825	$C_{21}H_{20}O_{13}$	-0.6	Myricetin 3-galactoside
91	6.05	463.0877	$C_{21}H_{20}O_{12}$	-0.5	Myricitrin
92	10.28	271.0606	$C_{15}H_{12}O_5$	-0.6	Naringenin
93	3.85	609.1255	$C_{29}H_{24}O_{12}$	0.5	Neotheaflavin
94	6.5	593.1509	$C_{27}H_{30}O_{15}$	-0.3	Nicotiflorin
95	4.93	777.1307	$C_{36}H_{28}O_{17}$	-0.2	Oolongtheanin
96	3.76	218.103	$C_9H_{17}NO_5$	-0.4	Pantothenic acid
97	7.09	163.0396	$C_9H_8O_3$	-0.4	p-coumaric acid
98	15.02	605.2408	$C_{35}H_{34}N_4O_6$	0.3	Phaeophorbide B
99	3.53	164.0713	$C_9H_{11}NO_2$	-0.4	Phenylalanine
100	15.68	591.2612	$C_{35}H_{36}N_4O_5$	-0.1	Pheophorbide A

101	2.65	125.0241	$C_6H_6O_3$	-0.3	Phloroglucinol
102	4.9	277.0713	$C_{14}H_{14}O_6$	-0.5	Planchol A
103	4.3	135.0447	$C_8H_8O_2$	-0.5	p-OH-acetophenone
104	4.4	577.1349	$C_{30}H_{26}O_{12}$	-0.2	Procyanidin B2
105	4.98	729.1463	$C_{37}H_{30}O_{16}$	0.2	Procyanidin B-2 3'-gallate
106	4.4	577.1349	$C_{30}H_{26}O_{12}$	-0.2	Procyanidin B3
107	4.4	577.1349	$C_{30}H_{26}O_{12}$	-0.2	Procyanidin B4
108	4.4	577.1349	$C_{30}H_{26}O_{12}$	-0.2	Procyanidin B5
109	4.53	865.1977	$C_{45}H_{38}O_{18}$	-0.8	Procyanidin C1
110	3.85	609.1255	$C_{30}H_{26}O_{14}$	0.5	Prodelphinidin B
111	3.82	153.019	$C_7H_6O_4$	-0.3	Protocatechuic acid
112	7.62	481.114	$C_{25}H_{22}O_{10}$	0	Puerin C
113	7.62	481.114	$C_{25}H_{22}O_{10}$	0	Puerin E
114	4.67	416.1347	$C_{21}H_{23}NO_8$	-0.4	Puerin V
115	4.67	416.1347	$C_{21}H_{23}NO_8$	-0.4	Puerin VI
116	4.67	416.1347	$C_{21}H_{23}NO_8$	-0.4	Puerin VII
117	4.67	416.1347	$C_{21}H_{23}NO_8$	-0.4	Puerin VIII
118	2.65	125.0241	$C_6H_6O_3$	-0.3	Pyrogallol
119	9.14	301.035	$C_{15}H_{10}O_7$	-0.4	Quercetin
113	7.62	481.114	$C_{25}H_{22}O_{10}$	0	Puerin E
114	4.67	416.1347	$C_{21}H_{23}NO_8$	-0.4	Puerin V
115	4.67	416.1347	$C_{21}H_{23}NO_8$	-0.4	Puerin VI
116	4.67	416.1347	$C_{21}H_{23}NO_8$	-0.4	Puerin VII
117	4.67	416.1347	$C_{21}H_{23}NO_8$	-0.4	Puerin VIII
118	2.65	125.0241	$C_6H_6O_3$	-0.3	Pyrogallol
119	9.14	301.035	$C_{15}H_{10}O_7$	-0.4	Quercetin
120	5.25	479.0825	$C_{20}H_{18}O_{11}$	-0.6	Quercetin 3-arabinopyranoside
121	6.8	447.0929	$C_{21}H_{20}O_{11}$	-0.4	Quercetin 3-rhamnoside
122	5.77	609.1462	$C_{27}H_{30}O_{16}$	0.1	Quercetin-3-robinobioside
123	0.8	191.0556	$C_7H_{12}O_6$	-0.5	Quinic acid
124	4.86	431.1917	$C_{19}H_{30}O_8$	-0.5	Roseoside
125	5.77	609.1462	$C_{27}H_{30}O_{16}$	0.1	Rutin
126	5.97	295.0815	$C_{13}H_{14}O_5$	-0.8	Salicifoliol
127	4.15	137.024	$C_7H_6O_3$	-0.4	Salicylic acid
128	4.31	173.045	$C_7H_{10}O_5$	-0.6	Shikimic acid
129	3.91	223.0605	$C_{11}H_{12}O_5$	-0.7	Sinapic acid
130	5.57	197.0451	$C_9H_{10}O_5$	-0.4	Syringic acid
131	4.49	349.058	$C_{15}H_{12}O_7$	1.5	Taxifolin
132	3.93	321.0612	$C_{14}H_{12}O_6$	-0.4	Teadenol A

133	3.93	321.0612	$C_{14}H_{12}O_6$	-0.4	Teadenol B
134	6.31	231.0656	$C_{13}H_{12}O_4$	-0.7	Teasperin
135	5.44	277.0711	$C_{14}H_{14}O_6$	-0.7	Teasperol
136	7.41	399.0722	$C_{20}H_{16}O_9$	0.1	Theaflagallin
137	4.62	565.1556	$C_{26}H_{30}O_{14}$	-0.7	Theaflavanoside I
138	4.92	897.1537	$C_{43}H_{32}O_{19}$	1.7	Theaflavate A
139	4.49	745.1406	$C_{36}H_{28}O_{15}$	-0.5	Theaflavate B
140	4.46	473.0722	$C_{21}H_{16}O_{10}$	-0.3	Theaflavic acid
141	9.56	867.1419	$C_{43}H_{32}O_{20}$	0.4	Theaflavin 3,3'-digallate
142	9.28	715.1304	$C_{36}H_{28}O_{16}$	0	Theaflavin-3'-gallate
143	9.28	715.1304	$C_{36}H_{28}O_{16}$	0	Theaflavin-3-gallate
144	4.75	913.1473	$C_{44}H_{34}O_{22}$	0.4	Theasinensin A
145	4.16	761.1363	$C_{37}H_{30}O_{18}$	0.3	Theasinensin B
146	3.85	609.1255	$C_{30}H_{26}O_{14}$	0.5	Theasinensin C
147	4.75	913.1473	$C_{44}H_{34}O_{22}$	0.4	Theasinensin D
148	3.85	609.1255	$C_{30}H_{26}O_{14}$	0.5	Theasinensin E
149	4.92	897.1537	$C_{44}H_{34}O_{21}$	1.7	Theasinensin G
150	3.46	343.0668	$C_{14}H_{16}O_{10}$	-0.3	Theogallin
151	6.97	179.0557	$C_7H_8N_4O_2$	-1.8	Theophylline
152	9.46	593.1299	$C_{30}H_{26}O_{13}$	-0.2	Tiliroside
153	3.95	203.0819	$C_{11}H_{12}N_2O_2$	-0.7	Tryptophan
154	1.75	180.0661	$C_9H_{11}NO_3$	-0.5	Tyrosine
155	4.07	167.0343	$C_8H_8O_4$	-0.7	Vanillic acid
156	6.5	593.1509	$C_{27}H_{30}O_{15}$	-0.3	Vicenin 2
157	5.92	431.0983	$C_{21}H_{20}O_{10}$	-0.1	Vitexin
158	6.5	593.1509	$C_{27}H_{30}O_{15}$	-0.3	Vitexin-2
159	6.16	577.1552	$C_{27}H_{30}O_{14}$	-1.1	Vitexin-2''-rhamnoside
160	6.5	593.1509	$C_{27}H_{30}O_{15}$	-0.3	Vitexin-4''-glucoside
161	1.65	151.0256	$C_5H_4N_4O_2$	-0.5	Xanthine
162	5.94	293.0663	$C_{13}H_{12}O_5$	-0.3	Xanthocerin
163	12.86	249.1851	$C_{15}H_{24}$	-1	α-Gurjunene
164	12.86	249.1851	$C_{15}H_{24}$	-1	α-Humulene
165	13.6	277.2168	$C_{18}H_{30}O_2$	-0.5	α-linolenic acid
166	12.86	249.1851	$C_{15}H_{24}$	-1	α-Muurolene
167	2.49	331.0668	$C_{13}H_{16}O_{10}$	-0.3	β-glucogallin
168	1.64	102.0558	$C_4H_9NO_2$	-0.2	γ-Aminobutyric acid
169	5.85	441.0825	$C_{22}H_{18}O_{10}$	-0.2	Epicatechin 3-gallate
170	3.78	305.0666	$C_{15}H_{14}O_7$	-0.1	Epigallocatechin
171	4.74	457.0775	$C_{22}H_{18}O_{11}$	-0.2	Epigallocatechin-3-gallate

172	4.3	179.0343	$C_9H_8O_4$	-0.7	Caffeic acid
173	4.3	353.0874	$C_{16}H_{18}O_9$	-0.4	Chlorogenic acid
174	5.87	300.9987	$C_{14}H_6O_8$	-0.3	Ellagic acid
175	7.6	193.05	$C_{10}H_{10}O_4$	-0.7	Ferulic acid
176	8.84	563.1195	$C_{29}H_{24}O_{12}$	0	Theaflavin
177	0.96	173.0926	$C_7H_{14}N_2O_3$	-0.5	Theanine
178	0.81	179.0556	$C_7H_8N_4O_2$	-1.8	Theobromine

Supplementary Table S4. HPLC detection of 19 amino acids in FBT samples（mg/g）

	S1	S2	S3	S4	S5	S6	S7
Asp	1.00±0.03b	1.08±0.06a	0.85±0.02c	0.29±0.003d	0.33±0.003d	ND	0.14±0.004e
Glu	1.25±0.17a	1.24±0.11a	1.00±0.03b	0.18±0.04c	0.03±0.02c	ND	ND
Asn	0.39±0.004b	0.37±0.02b	0.36±0.004b	0.28±0.05c	0.30±0.03c	0.36±0.001b	0.19±0.02d
Gln	0.36±0.01a	0.33±0.02a	0.37±0.01a	0.15±0.008c	0.17±0.01c	0.21±0.02b	0.18±0.07d
Gly	0.55±0.03b	0.67±0.03a	0.54±0.01b	0.45±0.01c	0.49±0.02c	0.65±0.03a	ND
Pro	0.58±0.06a	0.60±0.07a	0.59±0.002a	ND	ND	ND	ND
Ala	1.17±0.29a	1.30±0.07a	0.48±0.02b	0.46±0.02b	ND	ND	ND
Ser	0.28±0.07a	0.25±0.02ab	0.17±0.01c	0.27±0.07ab	0.20±0.008bc	ND	ND
L-The	6.36±0.53a	0.89±0.13b	0.92±0.05b	0.54±0.11bc	0.29±0.006c	0.25±0.12c	0.35±0.07c
Tyr	2.21±0.33b	0.60±0.07d	0.56±0.01d	3.33±0.41a	3.14±0.24a	1.28±0.12c	0.42±0.39d
Arg	0.97±0.11d	1.13±0.04cd	1.12±0.01d	1.43±0.02ab	1.55±0.16a	1.54±0.03a	1.31±0.14bc
Val	0.14±0.02d	ND	0.31±0.001c	0.68±0.02a	0.74±0.10a	0.53±0.10b	0.42±0.10bc
Met	0.02±0.006c	0.24±0.15b	0.31±0.02b	0.03±0.01c	ND	0.73±0.14a	0.24±0.09b
Trp	0.40±0.11a	0.35±0.11ab	0.24±0.02bc	0.15±0.01c	0.14±0.03c	ND	ND
Ile	0.12±0.05bc	0.09±0.03bc	ND	ND	ND	0.17±0.01ab	0.21±0.25a
Phe	0.14±0.004ab	0.18±0.02ab	0.20±0.02a	0.11±0.01c	0.09±0.01c	0.09±0.08bc	0.19±0.07ab
Leu	0.22±0.06a	0.09±0.02b	0.04±0.01bc	ND	0.01±0.01c	0.04±0.03bc	ND

Note: Values in the same column labeled with different superscript letters (a–e) differ significantly ($p < 0.05$).

Supplementary Table S5. VIF value of 9 critical ingredients

	TP	TPs	Gallic acid	Caffeine	EGC	GCG	Gln	L-The	Lys
VIF value	7.862	2.809	3.642	2.835	4.516	6.113	4.759	6.150	2.533

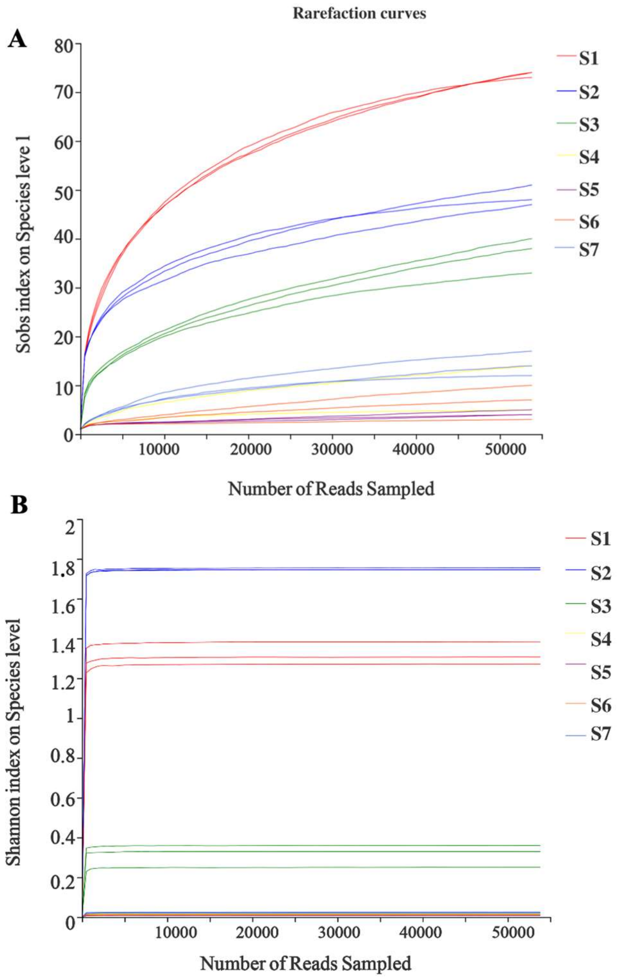

Supplementary Figure S1. Rarefaction curve analysis of the fungal community in the fermentation process of FBT. Fungus sobs curve (A), fungus Shannon-Wiener curve (B)

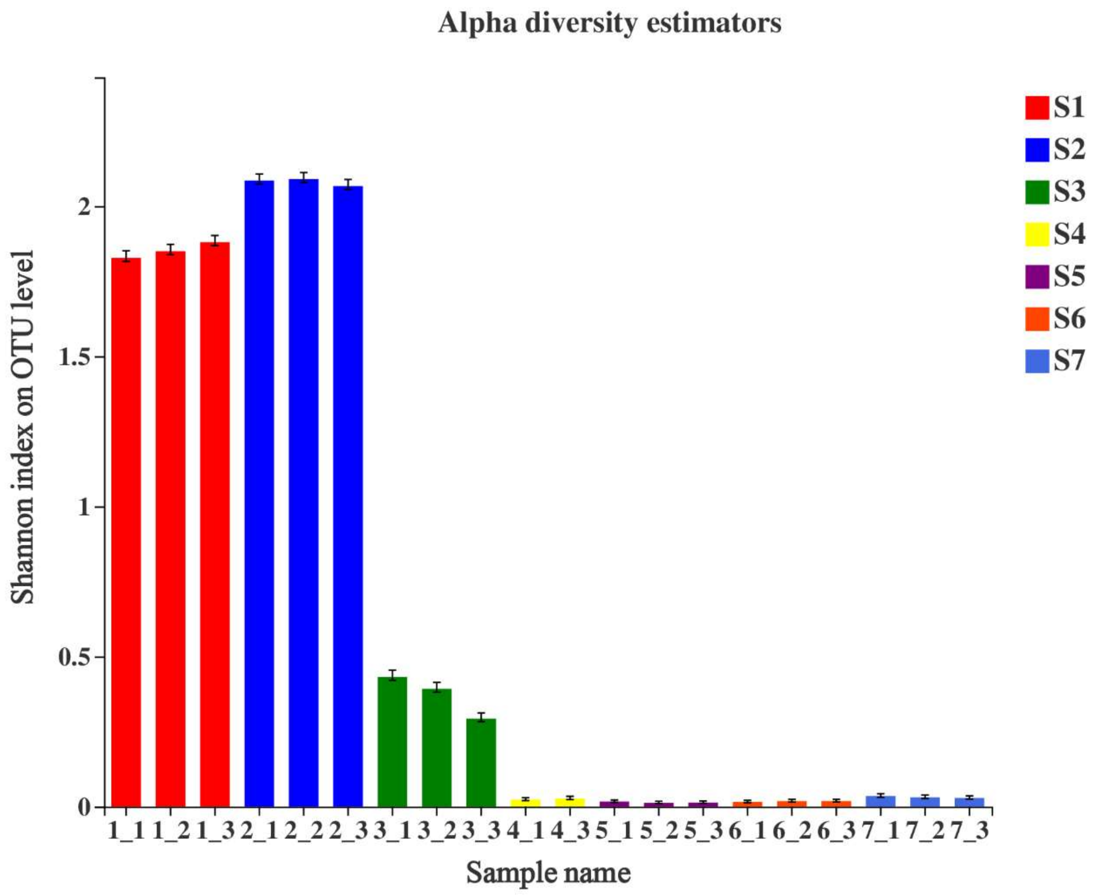

Supplementary Figure S2. The number of operational taxonomic units (OTUs) and alpha diversity estimators for fungal communities in FBT samples.

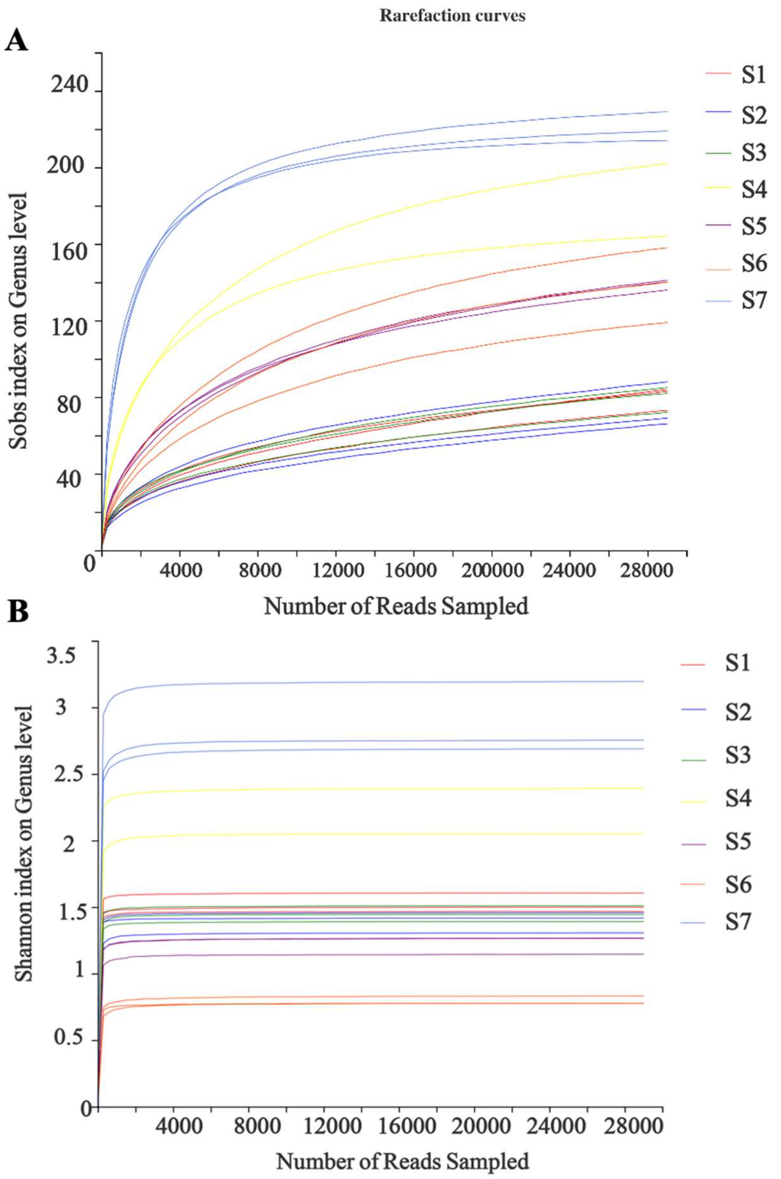

Supplementary Figure S3. Rarefaction curve analysis of the bacterial community in the fermentation process of FBT. Bacterial sobs curve (A), Bacterial Shannon-Wiener curve (B)

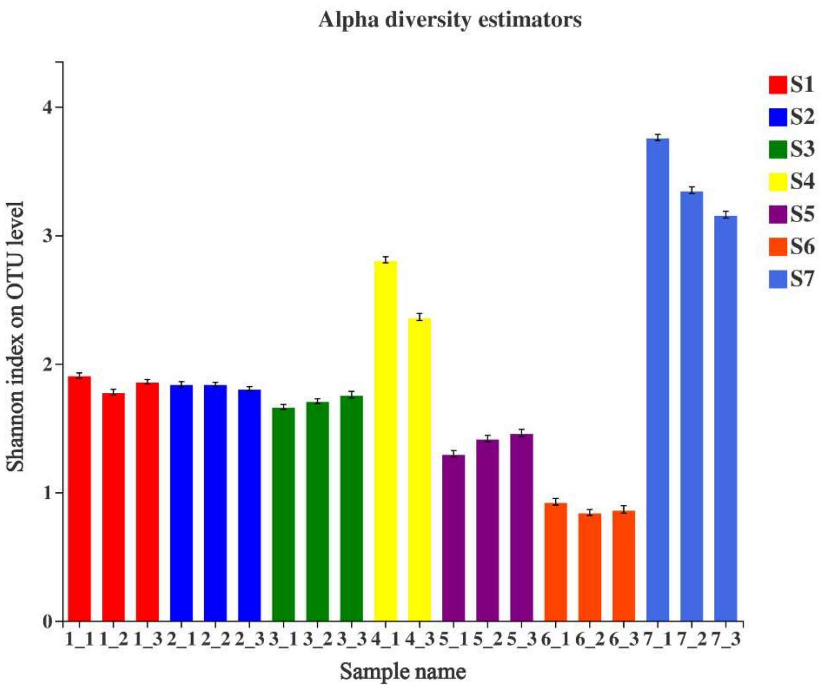

Supplementary Figure S4. The number of operational taxonomic units (OTUs) and alpha diversity estimators for bacterial communities in FBT samples.
